# The platelet-to-lymphocyte ratio reflects the severity of obstructive sleep apnea syndrome and concurrent hypertension

**DOI:** 10.1186/s40885-015-0036-3

**Published:** 2016-01-04

**Authors:** Yeo-Jeong Song, Jae Hwan Kwon, Joo Yeon Kim, Bo Young Kim, Kyoung Im Cho

**Affiliations:** 1 Division of Cardiology, Department of Internal Medicine, Kim-Hae Jung Ang Hospital, Busan, Korea; 2 Department of Otorhinolaryngology Head-Neck Surgery, Kosin University College of Medicine, Busan, Korea; 3 Department of Otorhinolaryngology Head-Neck Surgery, Maryknoll Medical Center, Busan, Korea; 4 Division of Cardiology, Department of Internal Medicine, Cardiovascular Research Institute, Kosin University School of Medicine, 34 Amnam-Dong, Seo-Ku, Busan, 602-702 Korea

**Keywords:** Platelet-to-lymphocyte ratio, Obstructive sleep apnea, Hypertension

## Abstract

**Background:**

Chronic intermittent hypoxia, platelet activation and inflammation all play roles in the pathogenesis of obstructive sleep apnea syndrome (OSAS), which may increase the risk of cardiovascular disease (CVD). The aim of this study was to evaluate the relationship of the platelet-to-lymphocyte ratio (PLR) as a new biomarker showing systemic inflammation and platelet distribution width (PDW) as an indicator of platelet activation to the severity of OSAS.

**Methods:**

A total of 290 patients suspected with OSAS who underwent a full night of polysomnography were included. The patients were placed into 4 separate groups according to their apnea-hypopnea index (AHI) scores; the control group (AHI <5), mild OSAS group (AHI 5–15), moderate OSAS group (AHI 16–30), and severe OSAS group (AHI >30). CVD risk was defined by the presence of hypertension, diabetes mellitus, current smoking, and dyslipidemia.

**Results:**

Higher AHI groups were significantly correlated with increasing age, body mass index, systolic blood pressure and male sex. PLR and PDW were also significantly associated with AHI (r = 0.417 for PLR and *r* = 0.227 for PDW, all *p*-values < 0.001) and the Epworth sleepiness scale (*r* = 0.160 for PLR and *r* = 0.189 for PDW, all *p*-values <0.05). Multivariate regression analysis revealed that AHI ≥9.2 (adjusted odds ratios [OR] 5.03, 95 % confidential interval (CI) = 1.67-15.2, *p* = 0.004) and PLR ≥159 (adjusted OR 2.81, 95 % CI = 1.34-5.91, *p* = 0.006) were independently associated with the presence of hypertension.

**Conclusion:**

PLR and PDW are associated with OSAS severity. PLR may also be useful as a systemic biomarker for the concurrent hypertension in OSAS patients.

## Background

Obstructive sleep apnea (OSA) induces hypoxia and hypercapnia, which adversely affect the myocardial oxygen supply. These changes ultimately contribute to the development of altered metabolic, immune, and inflammatory systemic responses [1]. Several studies have previously demonstrated that obstructive sleep apnea syndrome (OSAS) is an independent risk factor for cardiovascular morbidity and mortality [2, 3] including coronary artery disease and stroke [4, 5]. There is some evidence suggesting that OSAS is associated with low-levels of systemic inflammation and oxidative stress [1]. This chronic inflammation in OSAS may play an important role in the progression of atherosclerosis and cardiovascular disease (CVD) [6–9]. Therefore, it is important to clarify the association between inflammation and disease severity in order to determine the influence of OSAS on CVD.

Increased platelet activation plays a major role in the initiation and progression of atherosclerosis [10]. Recent studies have also introduced a new inflammatory marker, the platelet-to-lymphocyte ratio (PLR), which can predict adverse outcomes in CVD [11, 12]. Furthermore, interactions between platelets, leukocytes and endothelial cells during the inflammatory cascade may promote atherosclerotic changes [12–14]. During an inflammatory response, there is an increase in the platelet number and platelet swelling, both of which may affect the platelet distribution width (PDW) [15, 16]. The PLR and PDW are both relatively easy to measure as routine laboratory biomarkers of systemic inflammation. However, there is little data regarding their usefulness in patients with OSAS. Therefore, the aim of this study was to evaluate the relationship between PLR, PDW and OSAS severity, as assessed by polysomnographic parameters. In addition, we investigated the role of PLR in hypertension, and the association between PLR and AHI severity.

## Methods

### Patient selection

This is a retrospective cohort study performed at two centers. A total of 290 patients who had undergone polysomnography between January 2010 and December 2014 were evaluated. Patients were considered for inclusion if they were between 18 and 80 years old, had normal renal function and regular menstruation (with regard to the women included). Patients were excluded if any of the following criteria applied: systemic disease such as liver disease; neurologic disorders or malignancy; secondary hypertension; respiratory infection and failure; critical illness with poor functional status; refractory arrhythmia; history of heart failure; history of acute coronary syndrome, myocardial infarction and/or any revascularization procedure. Demographic characteristics including age, sex, height, weight, current medications, smoking history and other diseases were recorded at first visit. Patients were assessed for any CVD risk factors including the presence or absence of medically diagnosed hypertension, diabetes mellitus, dyslipidemia, and current smoking status. Blood pressure (BP) was measured with using a standard mercury manometer. Hypertension was defined as systolic BP (SBP) ≥140 mmHg, diastolic BP (DBP) ≥90 mmHg and/or current use of antihypertensive drugs. Body mass index (BMI) was calculated as the ratio of dry weight in kilograms to height in meters squared. This study was approved by the Institutional Review Board.

### Polysomnography

The full night polysomnography (PSG) studies used for OSAS diagnosis were retrospectively reviewed. Full night PSG (Embla N7000, Broomfield CO, USA) examination was performed using a device equipped with a snoring sensor, respiratory effort sensors, pressure sensor, body position center, thermister, electroencephalography (EEG), electrocardiogram (ECG), pulse oximeter with oxygen saturation, lower extremity electromyography and chest excursion. The value index of PSG testing included AHI scores, percentage of sleep efficacy, total sleep time, baseline/lowest oxygen saturation, and the Epworth sleepiness scale (ESS). The ESS was evaluated before PSG using a survey. Apnea was defined as a standstill in the airflow for more than 10 s. Hypopnea was defined as ≥30 % reduction in airway flow for more than 10 s followed by a decrease of approximately 4 % in the patient’s oxygen saturation [17]. AHI was used as an index to represent the severity of obstructive sleep apnea. It is defined as the average frequency of apnea and hypopnea events per hour during sleep. The subjects were distributed into four groups according to their AHI scores: the control group with AHI <5, mild OSAS group with AHI 5–15, moderate OSAS group with AHI 16–30, and severe OSAS group with AHI score >30.

### Laboratory measurements

The biochemical parameters analyzed included total cholesterol, triglyceride, fasting glucose, erythrocyte sedimentation rate (ESR), high sensitivity C-reactive protein (hs-CRP), and complete blood cell count. The PLR was calculated by dividing the lymphocyte from the platelet.

### Statistical analyses

Statistical analyses were performed using the commercially available computer program SPSS 18.0 for Windows (SPSS Inc., Chicago, IL, USA). Data are presented as means ± standard deviations for continuous variables and their percentages (%) for categorical data. The Mann - Whitney *U* test was used for continuous variables and the chi-square test was used for categorical data. The Kolmogorov-Smirnov test was used to test the data’s normality. Parameter differences among the three groups were evaluated using a one-way analysis of variance (ANOVA) or the Kruskal–Wallis test for normally or non-normally distributed variables, respectively. The Tukey test was used for multiple comparisons. Pearson correlation coefficients were used to determine relationships between variables. Linear association analysis was used to verify the correlations between variables. Receiver operator characteristic (ROC) curve analysis was used to estimate the cutoff values for AHI and PLR in the prediction of hypertension. Multivariate logistic regression models were built to determine which variables are independently associated with concurrent hypertension. Two-tailed *p-values* < 0.05 were considered statistically significant.

## Results

**Fig. 1:**
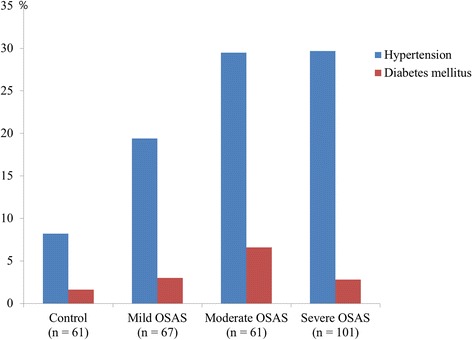
The prevalences of hypertension and diabetes mellitus were significantly higher in the moderate/severe OSAS group than they were in the control group

**Fig. 2:**
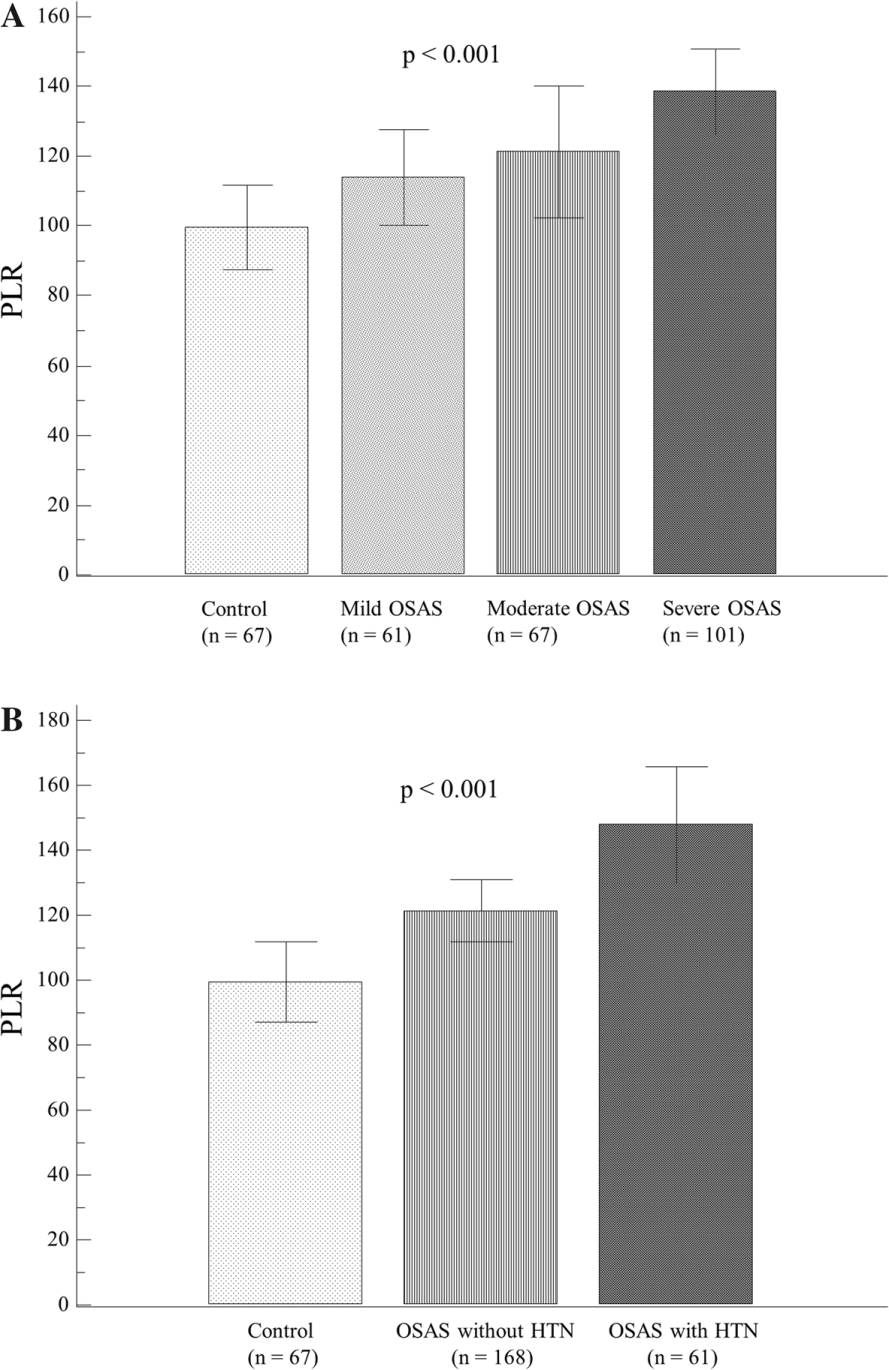
The relationship between platelet-to-lymphocyte ratio (PLR) and the clinical features of obstructive sleep apnea syndrome (OSAS). **a** There were gradual but significant increases in the PLR (controls: 99.5 ± 42.1, mild OSAS; 113.8 ± 45.2, moderate OSAS; 121.3 ± 62.9 and severe OSAS; 138.6 ± 59.9, *p* = 0.001). **b** The PLR was most significantly elevated in OSAS patients with hypertension compared to those without and to controls

There was a total 290 patients including 193 men (66.6 %) and 97 women (33.4 %) included in this study. The mean age was 48.6 ± 13.4 years (range: 18–78). There were 61 control patients, 67 mild OSAS, 61 moderate OSAS, and 101 severe OSAS patients. Table 1 shows the baseline clinical and laboratory characteristics of each group. In general, the OSAS groups had older patients with significantly more men than did the control group. In addition, the BMI, SBP, heart rate, and fasting blood glucose were higher in the OSAS groups than the control group. Age, BMI, and male proportion increased significantly with increasing AHI. The prevalence of hypertension or diabetes mellitus was significantly higher in the moderate/severe OSAS group than they were in the control group (all *p*-values < 0.05, Fig. 1). In addition, as the OSAS severity increased, there were significant gradual increases in PLR (controls: 99.5 ± 42.1, mild OSAS; 113.8 ± 45.2, moderate OSAS; 121.3 ± 62.9 and severe OSAS; 138.6 ± 59.9, *p* = 0.001, Fig. 2a) and in PDW (controls: 15.9 ± 1.12, mild OSAS; 16.3 ± 0.95, moderate OSAS; 16.4 ± 0.99 and severe OSAS; 16.5 ± 0.82, *p* = 0.003). Table 2 shows the polysomnography data among the 4 groups. With increasing AHI, there were significant stepwise decreases in the total sleep time and the saturation value, and significant increase in ESS. As expected, there were significant differences between the AHI groups with regard to the pre ESS score, baseline saturation and lowest saturation (all *p*-values <0.05).

**Table 1:** Baseline patient characteristics

	Control (*n* = 61)	Mild OSAS (*n* = 67)	Moderate OSAS (*n* = 61)	Severe OSAS (*n* = 101)	ANOVA *p*
Age, years	44.0 ± 15.4	48.2 ± 13.4	49.1 ± 11.8	51.4 ± 12.5^*^	0.003
Male, *n* (%)	33 (54.1)	39 (58.2)	41 (67.2)^*^	80 (79.2)^*^	<0.001
BMI, kg/m^2^	24.2 ± 4.43	24.7 ± 3.20	26.4 ± 3.58^*,+^	26.9 ± 3.41^*,+^	<0.001
SBP, mmHg	123.4 ± 13.7	123.5 ± 13.1	128.5 ± 13.6^*^	128.2 ± 12.4^*^	0.027
DBP, mmHg	78.4 ± 10.7	77.0 ± 8.9	80.8 ± 9.2	81.2 ± 9.9^*^	0.039
Heart rate, /min	72.6 ± 7.90	73.3 ± 10.3	76.3 ± 10.5	76.7 ± 9.15^*^	0.020
Hypertension, *n* (%)	5 (8.2)	13 (19.4)	18 (29.5)^*^	30 (29.7)^*^	0.005
Diabetes mellitus, *n* (%)	1 (1.6)	2 (3.0)	4 (6.6)	13 (12.9) ^*^	0.048
Dyslipidemia, *n* (%)	2 (3.3)	2 (3.0)	3 (4.9)	3 (3.0)	0.917
Smoking, *n* (%)	6 (9.8)	14 (20.9)	12 (19.7)	18 (17.8)	0.123
Total cholesterol, mg/dl	170.0 ± 38.2	184.0 ± 34.6	190.6 ± 45.6	172.4 ± 46.9	0.161
TG, mg/dl	141.1 ± 84.8	151.2 ± 8.03	160.8 ± 89.1	176.1 ± 144.9	0.606
FBS, mg/dl	101.5 ± 17.2	92.8 ± 10.3	105.8 ± 21.7	111.6 ± 45.3	0.034
ESR, mm/hr	7.8 ± 11.6	17.9 ± 30.2	15.5 ± 15.3	14.0 ± 10.9	0.224
hs-CRP, mg/L	0.53 ± 1.32	1.14 ± 2.28	0.82 ± 1.38	1.12 ± 2.08	0.529
WBC, x10^6^/L	6759 ± 1806	7283 ± 2031	7314 ± 2976	7724 ± 2051	0.103
Neutrophil, %	53.1 ± 11.2	59.1 ± 11.0	55.1 ± 13.8	60.5 ± 10.3^*^	0.001
Lymphocyte, %	36.1 ± 9.87	33.0 ± 12.8	30.0 ± 9.22	27.4 ± 8.35^*^	<0.001
Eosinophil, %	2.24 ± 1.66	3.01 ± 2.03	2.60 ± 1.99	2.90 ± 2.16	0.217
Monocyte, %	7.91 ± 2.54	7.44 ± 2.15	8.51 ± 5.96	7.35 ± 1.71	0.234
RBC, x10^9^/L	4587 ± 423.9	4692 ± 377.2	4660 ± 282.4	4599 ± 378.1	0.286
Hemoglobin, g/dl	13.5 ± 1.5	14.0 ± 1.4	13.8 ± 1.6	14.4 ± 1.6^*^	0.004
Hematocrit, %	40.2 ± 4.4	41.2 ± 4.2	40.2 ± 6.6	42.5 ± 4.5^*^	0.010
RDW, %	13.5 ± 1.57	13.3 ± 1.25	13.5 ± 1.05	13.3 ± 0.76	0.735
Platelet, x10^9^/L	216.7 ± 54.4	224.8 ± 56.0	231.3 ± 80.6	252.4 ± 66.8^*^	0.007
PLR	99.5 ± 42.1	113.8 ± 45.2	121.3 ± 62.9^*^	138.6 ± 59.9^*^	0.001
PDW, %	15.9 ± 1.12	16.3 ± 0.95	16.4 ± 0.99^*^	16.5 ± 0.82^*^	0.003

All values are presented as means ± SD. BP, blood pressure; hs-CRP, high sensitivity C-reactive protein; OSAS: obstructive sleep apnea syndrome; BMI, body mass index; SBP, systolic blood pressure; DBP, diastolic blood pressure; TG, triglyceride; FBS, fasting blood sugar; ESR, erythrocyte sedimentation rate; WBC, white blood cell; RBC, white blood cell; RDW, red cell distribution width; PLR, platelet lymphocyte ratio; PDW, platelet volume distribution width, *p < 0.05 vs. control group, ^+^ *p* < 0.05 vs. mild OSAS group

**Table 2:** Group comparison according to polysomnographic parameters

	Control (*n* = 61)	Mild OSAS (*n* = 67)	Moderate OSAS (*n* = 61)	Severe OSAS (*n* = 101)	ANOVA *p*
AHI	1.91 ± 1.69	9.14 ± 3.03^*^	21.5 ± 4.98^*,+^	72.5 ± 51.7^*,+^	<0.001
Sleep efficiency, %	81.0 ± 9.14	81.0 ± 9.91	82.0 ± 10.3	77.1 ± 13.1^*^	0.026
Total sleep time, min	322.8 ± 44.8	298.7 ± 60.4	292.2 ± 62.4	257.0 ± 78.2^*^	<0.001
Stage 1, min	58.2 ± 32.0	39.5 ± 37.9	48.2 ± 53.7	73.5 ± 59.8^*^	0.006
Stage 2, min	139.6 ± 71.8	103.2 ± 67.3	125.7 ± 69.6	70.9 ± 43.30^*^	<0.001
Baseline SaO_2_, %	97.3 ± 0.96	95.5 ± 1.77	93.9 ± 3.07	92.0 ± 4.87^*^	<0.001
Lowest SaO_2,_ %	91.3 ± 3.09	88.4 ± 3.82	81.3 ± 8.68^*^	79.0 ± 8.87^*^	<0.001
ESS	8.7 ± 4.6	8.9 ± 5.2	10.4 ± 5.9	11.6 ± 6.0^*^	0.029

AHI: apnea-hypopnea index; ESS: Epworth sleepiness scale

*p < 0.05 vs. control group, +*p* < 0.05 vs. mild OSAS group

**Fig. 3:**
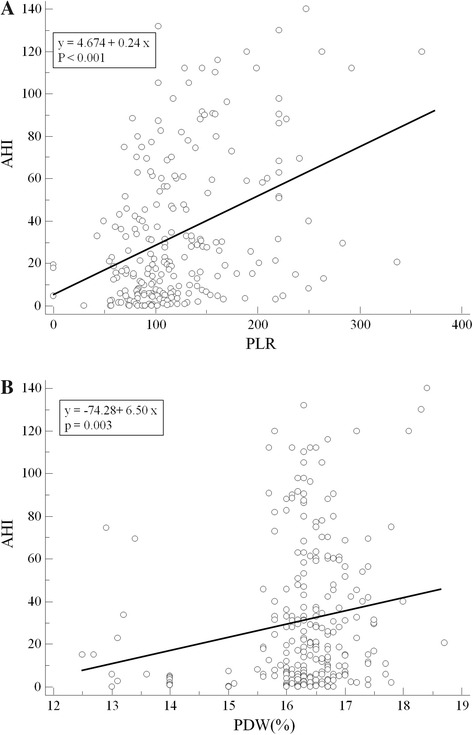
The correlations between inflammatory markers and clinical factors of OSAS. **a** The platelet to lymphocyte ratio (PLR) was significantly associated with the apnea-hypopnea index (AHI) (*r* = 0.417, *p* <0.001). **b** The platelet distribution width (PDW) was significantly correlated with AHI (*r* = 0.227, *p* <0.001)

Then, we analyzed the correlations between inflammatory markers and the clinical OSAS factors. PLR was significantly associated with AHI (*r* = 0.417, *p* <0.001, Fig. 3a), minimum O_2_ saturation values (*r* = −0.192, *p* = 0.005), ESS (*r* = 0.160, *p* = 0.047) and hs-CRP (*r* = 0.367, *p* < 0.001). In addition, PDW was significantly correlated with AHI (*r* = 0.227, *p* <0.001, Fig. 3b), minimum O_2_ saturation values (*r* = −0.165, *p* = 0.011) and ESS (*r* = 0.189, *p* = 0.018). However, red blood cell count (RBC) showed no significant correlation with AHI (*r* = −0.103, *p* = 0.081). Multivariate logistic regression analysis showed that PLR (beta = 0.358, *p* <0.001) and PDW (beta = 0.186, *p* = 0.002) were independently associated with AHI after adjustment of the presence of hypertension, diabetes mellitus, dyslipidemia, obesity defined as BMI ≥ 25 kg/m^2^ and current smoking status.

In sub-group analysis, OSAS patients with hypertension were found to have significantly higher AHI, ESS, PLR and PDW values than do those without hypertension (Table 3). Among these parameters, PLR and AHI were most significantly elevated in OSAS patients with hypertension compared to those without and to controls (Fig. 2b, Table 3). Furthermore, hypertension was significantly associated with AHI ≥9.2 (ROC area under curve: 0.66, 95 % confidence interval (CI) = 0.60–0.71, *p* < 0.001) and PLR ≥159 (ROC area under curve: 0.66, 95 % CI = 0.59–0.72, *p* < 0.001). Multivariate logistic regression analysis showed that AHI ≥9.2 (adjusted odds ratios [OR] 5.03, 95 % CI 1.67-15.2, *p* = 0.004) and PLR ≥159 (adjusted OR 2.81, 95 % CI = 1.34-5.91, *p* = 0.006) were independently associated with the concurrent hypertension in OSAS after adjusting for age, male gender, and BMI (Table 4).

**Table 3:** OSAS patient characteristics according to the presence of hypertension

	Control (*n* = 61)	OSAS without HTN (*n* = 168)	OSAS with HTN (*n* = 61)	ANOVA *p*
Age, years	44.0 ± 15.4	48.9 ± 13.0^*^	52.4 ± 11.4^*^	0.002
Male, *n* (%)	33 (54.1)	115 (68.5)	45 (73.8)	0.051
BMI, kg/m^2^	24.2 ± 4.4	26.0 ± 3.5^*^	26.6 ± 3.1^*^	0.001
SBP, mmHg	123.4 ± 13.7	125.7 ± 12.7	130.1 ± 13.6^*^	0.018
DBP, mmHg	78.4 ± 10.7	78.8 ± 9.0	82.4 ± 10.1^*^	0.031
Heart rate, /min	73.3 ± 10.3	74.4 ± 9.4	72.0 ± 9.2	0.177
Diabetes mellitus, *n* (%)	4 (6.5)	9 (5.4)	7 (11.5)	0.255
Dyslipidemia, *n* (%)	2 (3.3)	6 (3.6)	2 (3.3)	0.993
Smoking, *n* (%)	6 (9.8)	32 (19.0)	14 (23.0)	0.120
Total cholesterol, mg/dl	170.0 ± 38.2	186.1 ± 44.9	169.0 ± 38.6	0.082
TG, mg/dl	151.2 ± 80.3	157.5 ± 110.1	174.1 ± 128.3	0.716
FBS, mg/dl	101.5 ± 17.2	103.9 ± 33.7	109.9 ± 35.9	0.513
ESR, mm/hr	7.8 ± 11.6	16.4 ± 19.3	12.4 ± 11.2	0.095
hs CRP, mg/L	0.53 ± 1.32	1.02 ± 2.10	1.07 ± 1.61	0.427
PLR	99.5 ± 42.3	121.4 ± 55.5^*^	147.9 ± 61.8^*^	<0.001
PDW, %	15.9 ± 1.12	16.37 ± 0.81^*^	16.44 ± 1.13^*^	0.002
AHI	1.91 ± 1.69	33.8 ± 30.1^*^	58.2 ± 68.3^*^	<0.001
ESS	8.65 ± 4.64	10.1 ± 5.85^*^	12.4 ± 5.40^*^	0.009

BMI, body mass index; SBP, systolic blood pressure; DBP, diastolic blood pressure; HR: heart rate; TG: triglyceride; FBS: fasting blood sugar; ESR, erythrocyte sedimentation rate; hs-CRP, highly sensitive C-reactive protein; PLR, platelet lymphocyte ratio; PDW, platelet volume distribution width; AHI, apnea-hypopnea index; ESS, Epworth sleepiness scale; *p < 0.05 vs. control group

**Table 4:** Binary regression analysis of hypertension

Parameters	Odd ratio (95 % CI)	*p*-value	Adjusted Odd ratio (95 % CI)	*p*-value
Age, years	1.03 (1.01 to 1.05)	0.011	1.02 (0.99 to 1.05)	0.081
BMI, kg/m^2^	1.06 (0.99 to 1.14)	0.103	1.00 (0.90 to 1.10)	0.949
Male gender (%)	1.21 (0.67 to 2.18)	0.538	1.31 (0.57 to 2.84)	0.554
AHI ≥9.2	5.45 (2.38 to 12.48)	<0.001	5.03(1.67 to 15.2)	0.004
PLR ≥159	3.37 (1.67 to 6.79)	0.001	2.81 (1.34 to 5.91)	0.006

CI, confidence intervals, BMI, body mass index; AHI, apnea-hypopnea index; PLR, platelet lymphocyte ratio

## Discussion

In the present study, we showed that the indicator of platelet activation (PDW) and systemic inflammation (PLR) were both found to be significantly associated with OSAS severity (reflected by AHI). In addition, we have demonstrated that PLR may be a systemic biomarker that is independently associated with concurrent hypertension in OSAS patients. OSAS is a chronic disease that is frequently associated with various comorbidities including cognitive impairment, CVD, pulmonary disease, endocrine dysfunction, and neuropsychiatric problems [18–20]. The exact mechanism of OSAS and its subsequent comorbidities is not known. However, chronic systemic inflammation has been proposed in several recent studies as a possible mechanism [21–23]. Although the inflammation plays an important role in the progression and destabilization of atherosclerosis and CVD [24, 25], it is unclear if this it is the cause or result of OSAS. There have been conflicting reports on the relationship between inflammatory markers and the degree of the upper airway obstruction [26, 27]. Korkmaz M. et al. [26] showed that the traditional inflammatory markers such as CRP and ESR cannot predict the severity of the level of systemic inflammation in OSAS patients. Because systemic inflammation and resultant vascular insult is an important underlying mechanism in OSAS, exploring new inflammatory markers in OSAS may offer more information in order to predict the risk of associated diseases.

The PLR combines the predictive risk of platelet and lymphocyte counts into a new prognostic marker of inflammation for many types of cardiovascular disease including hypertension, coronary artery disease, occlusive peripheral arterial disease [11, 12], and atherosclerosis [28]. Elevated platelet counts reflect underlying inflammatory states, because platelets are acute phase reactants that are produced in response to various stimuli including systemic infections, inflammatory conditions, bleeding, and tumors. Low levels of lymphocytes represent an uncontrolled inflammatory pathway. Therefore, an elevated PLR is a useful inflammatory marker because it reflects the increase in platelet count, and decrease in lymphocyte count in an inflammatory state [29]. In the present study, we selected PLR as a potential inflammatory marker because it is easily calculated using the results of routine peripheral blood tests, and reflects systemic inflammation in OSAS patients. In addition, PDW increases with changes in platelet morphology and size, both of which occur in inflammatory states [16]. We hypothesized that both PLR and PDW would linearly increase according to the severity of OSAS. Both of these responses were demonstrated in this study, and because RBC showed no significant correlation with AHI, the correlation between PLR or PDW and AHI may not simply a consequence of increased blood viscosity in patients with OSAS. Moreover, although the PLR and hs-CRP showed significant correlation in our study, the useful inflammatory markers in OSAS were platelet-related inflammatory markers, not hs-CRP. In addition, inflammatory markers are also connected to the development and/or progression of obesity, type 2 diabetes mellitus, and hypertension. To explore the independent role of PLR and PDW on the severity of OSA, we performed multivariate logistic regression analysis, which showed that PLR and PDW were independently associated with AHI after adjustment of traditional CVD risk factors and obesity.

Another interesting finding from this study is that OSAS patients with concurrent hypertension had significantly higher PLR values than did those without hypertension. The hypertension that occurs with OSAS may be multifactorial in origin. Systemic inflammation likely plays a major role in the development of hypertension in OSAS. A previous study showed that OSAS patients with hypertension had higher levels of inflammation and insulin resistance than do those without hypertension [30]. Since systemic inflammation plays an important role in hypertension in OSAS, PLR may have a role in the development of hypertension in OSAS patients. We hypothesized that PLR, as a measurable biomarker of systemic inflammation, may be independently associated with hypertension in OSAS patients. Our results suggest that PLR may be useful inflammatory biomarker associated with concurrent hypertension in OSAS, even after adjusting for AHI. This finding suggests that inflammation and subsequent hypertension in OSAS are independent of the severity of upper way obstruction.

This study has several limitations. Since this was a retrospective cohort study, the blood sampling and test parameters were not performed with identical methods. Any variability may have influenced the value of the parameter. In addition, PLR and PDW were based on single measurements. It would be important to determine whether PLR changes over time, and if it is a consistent predictor of OSAS severity. Our study was also conducted with a relatively small sample of patients that was likely insufficient to demonstrate cardiovascular outcomes. In order to achieve more statistical power and substantiate our findings, larger randomized controlled trials are necessary.

## Conclusion

In conclusion, this study demonstrates evidence of inflammation and platelet activation in OSAS. Therefore, PLR and PDW may be relevant inflammatory markers of OSAS severity. Furthermore, PLR may be a useful inflammatory biomarker associated with concurrent hypertension in OSAS. Further, larger studies using PLR and PDW are needed to assess the effectiveness of continuous positive airway pressure therapy on OSAS.
